# Double burdened yet resilient: quality of life of caregivers of people with X-linked hypophosphatemia

**DOI:** 10.1093/jbmrpl/ziaf078

**Published:** 2025-04-30

**Authors:** Rafael Pinedo-Villanueva, Njoki Njuki, Maria T Sanchez-Santos, Muhammad Kassim Javaid

**Affiliations:** Nuffield Department of Orthopaedics, Rheumatology and Musculoskeletal Sciences, University of Oxford, Oxford, OX3 7HE, United Kingdom; National Institute for Health Research (NIHR) Oxford Biomedical Research Centre, University of Oxford, Oxford, OX3 9DU, United Kingdom; Nuffield Department of Orthopaedics, Rheumatology and Musculoskeletal Sciences, University of Oxford, Oxford, OX3 7HE, United Kingdom; Nuffield Department of Orthopaedics, Rheumatology and Musculoskeletal Sciences, University of Oxford, Oxford, OX3 7HE, United Kingdom; Nuffield Department of Orthopaedics, Rheumatology and Musculoskeletal Sciences, University of Oxford, Oxford, OX3 7HE, United Kingdom; National Institute for Health Research (NIHR) Oxford Biomedical Research Centre, University of Oxford, Oxford, OX3 9DU, United Kingdom

**Keywords:** X-linked hypophosphatemia, caregiver, quality of life, CarGOQoL, EQ-5D

## Abstract

X-linked hypophosphatemia (XLH) is a rare genetic disorder that leads to rickets, osteomalacia, and other skeletal abnormalities. Many people with XLH get support from informal caregivers, often family members, to help with their daily living. Although generally rewarding, being a caregiver can be associated with extra physiologic, physical, and mental health burdens, which are poorly researched and understood. This study aims to investigate the quality of life of people with XLH who are also caregivers of relatives with XLH. To do this, we conducted a cross-sectional study to characterize the quality of life of caregivers using both a caregiver-specific and a generic quality of life questionnaire, examined the association between the instruments for caregivers, and compared the generic quality of life between caregivers and matched non-caregivers. We used data from the Rare UK Diseases Study (RUDY), whose platform allows people with XLH to record their own characteristics and outcome measures, including the caregiver oncology quality of life questionnaire (CarGOQoL) and the generic EQ-5D. Caregivers and non-caregivers with XLH were matched on gender and age. Caregivers (*n* = 13) report not feeling burdened or seeing their self-esteem impacted by providing care, but their private life, leisure, and psychological well-being were affected. They report worse quality of life than the UK general population. CarGOQoL and EQ-5D were highly correlated (*p* < .001). Caregivers of people with XLH reported better quality of life than non-caregivers in all EQ-5D dimensions except for Anxiety/Depression (EQ-5D score of 0.467 vs 0.356 for non-caregivers). Our findings must be interpreted with caution, given the small sample size, although they are consistent with the literature. Caregivers play an important role in supporting the everyday life of those they look after. This role should be recognized, and helpful information and tools made available to support them in that process.

## Introduction

X-linked hypophosphatemia (XLH) is characterized by lifelong hypophosphatemia caused by excess circulating levels of FGF23 due to loss-of-function mutations in the *PHEX* gene. This leads to lifelong renal phosphate wasting. The physical manifestations in adults with XLH include chronic musculoskeletal pain and stiffness, short stature, lower limb deformities (often resulting from rickets in childhood, exposing long bones to abnormal biomechanical stress), abnormal gait, fractures and pseudofractures due to osteomalacia, accelerated osteoarthritis, dental abscesses, enthesopathy, impaired mobility and physical function, muscle function deficits, and acquired joint malalignment. All of these contribute to overall physical function worse than the general population and reduced health-related quality of life.^1^^,^^2^

As a result, many people diagnosed with XLH, as well as those with other debilitating rare diseases, get support from informal (unpaid) caregivers in many aspects of their daily living, such as with mobility^3^ and attending healthcare appointments.^4^ Given the mechanism of inheritance, caregivers are often, although not exclusively, family members.

Being a caregiver has been associated with physiologic, physical, and mental health outcomes as well as self-reported health, which have been linked to the care provided, duration, level of disability of the recipient, and even finances and family conflict, all acting as stressors generating mental and physical distress on the caregivers.^5^ Caregivers also have been found to report an increased mortality (RR = 1.63) after adjusting for sociodemographic factors, prevalent disease, and subclinical cardiovascular disease.^6^ The authors also show that not all caregivers report the mental and emotional strains associated with the higher mortality risk. Not all caregivers have such a negative experience; a survey of long-term care in the United States reported that the vast majority of caregivers associated their role with a positive experience.^7^

The published literature has primarily concentrated on the clinical aspects of XLH and the caregiver burden more generally or in the context of other diseases. Despite the critical role that caregivers play in helping people with XLH manage their symptoms and live their everyday life, no study has reported on the quality-of-life impact experienced by caregivers of people with XLH. Further, with XLH being an inherited rare disease, the negative impact of caregiving would generally come on top of the burden of the disease itself if the caregiver is a family member who also has XLH. Understanding the quality of life of caregivers is essential for developing comprehensive care strategies that support both people with XLH, their families, and their caregivers.

This study aims to describe the quality of life of people with XLH who are also caregivers of relatives with XLH using a caregiver-specific and a generic quality of life questionnaire. The study focused on 3 specific objectives: (1) to characterize quality of life of caregivers using both instruments; (2) to examine the association between the 2 instruments; and (3) to compare the generic quality of life findings between caregivers and non-caregivers. By investigating various dimensions of their well-being, including physical health, emotional stress, social interactions, and financial strain, this research seeks to provide a holistic view of the caregiving experience. The findings will contribute to support systems designed to improve the overall quality of life for caregivers, ultimately enhancing the care provided to people with XLH.

## Materials and methods

### Source of data

Patient-level data were extracted from the Rare UK Diseases Study (RUDY). RUDY is a fully online platform managed by the University of Oxford, featuring online registration and dynamic consent to facilitate the process of participants entering a wide range of data, including their own characteristics and various patient-reported outcomes (PROMs). RUDY is open to any individual with a rare disease that happens in less than 1 per 2000 people in the United Kingdom, as well as their blood relatives and partners. A key quality of this online study is that patients were and continue to be significantly involved in its creation, design, and management, which has helped improve recruitment and retention of participants, reduce clinical and researcher time entering data, and enable a function for participants to print off their data for personal use.^8^

### Quality of life of measures

Two questionnaires were used, one to capture caregiver burden completed only by caregivers, and a second measuring generic quality of life completed by both caregivers and non-caregivers.

To identify the most appropriate instrument reflecting the burden experienced by caregivers of people with XLH, a literature search was conducted to find instruments previously used. Instruments, caregiver domains, and their contributors were extracted. Domain relevance was assessed by online or phone interviews with 5 caregivers of people with XLH who volunteered from the RUDY Study. The PROQOLID database was searched to identify relevant instruments, which were then assessed according to whether they addressed the most important domains identified by the caregivers. Pre-selected instruments were then shared and discussed to select the most appropriate.

Twenty-seven publications were identified using 29 different instruments covering at least 17 distinct domains of burden on caregivers of people with musculoskeletal diseases, as none were identified specifically for caregivers of people with XLH. The 5 volunteer caregivers found *financial strain or difficulties* and *feeling of overall burden* to be the most impactful, followed by *stress* and *emotional strain or difficulties*. Fifty instruments were identified in PROQOLID and reviewed to assess whether they addressed these domains. Three instruments included all 4 domains: caregiver strain index, caregiver well-being scale, and caregiver oncology quality of life questionnaire (CarGOQoL). These were discussed with the volunteer caregivers and they selected CarGOQoL as the most appropriate instrument to capture the burden experienced by caregivers of people with XLH.

The CarGOQoL was developed as a specific quality of life questionnaire for caregivers of people with cancer based on the perspective of caregivers. It is composed of 29 items grouped into 10 dimensions: psychological well-being, burden, relationship with health care, administration and finances, coping, physical well-being, self-esteem, leisure time, social support, and private life.^9^ All dimensions were found to be relevant by the volunteer caregivers when assessing the quality of life of those who provide care and support for people with XLH. Items and domains are shown in Table S1 in the Supplementary documentation, with participants meant to answer each question based on what they “thought or felt during the last four weeks.” A license was obtained to add the questionnaire to the RUDY platform to conduct this study (CarGOQoL contact information and permission to use: Mapi Research Trust, Lyon, France, https://eprovide.mapi-trust.org).

Each item was assessed on a 5-point Likert Scale, ranging from 1 = “Never/Not at all” to 5 = “Always/Very Much,” with domain scores calculated by computing the mean of all item scores for that domain and linearly transforming it to a standardized score between 0 and 100. Item reversion (where, eg, a score of “1 - Never” was associated with better instead of worse quality of life) was applied to 19 of the 29 items (see Table S1 in the Supplementary documentation). A CarGOQoL overall score was calculated in an analogous manner by producing a standardized 0-100 score from the mean of the 10 domain scores. Higher scores indicate better quality of life.

For generic quality of life, we used the EuroQuol (EQ) 5D-5L questionnaire.^10^ The EQ-5D has 2 sections; on the first (descriptive), participants complete questions corresponding to the 5 dimensions of mobility, self-care, usual activities, pain/discomfort, and anxiety/depression, each assessed on a 5-point Likert Scale ranging from 1 = “No problem” to 5 = “Extreme problems” as they are on the day they complete it. Answers to these 5 questions can furthermore be converted into a score by applying the social valuation of health states by the general UK population. As per NICE recommendation,^11^ when using the EQ-5D-5L the utility score should be calculated by mapping the responses to the questions in the descriptive section onto the 3L value set using a published mapping function.^12^ The EQ-5D score is anchored at 0, representing death, and has a maximum value of 1, representing *perfect health*, with negative values allowed and representing health states worse than death. The second section of the EQ-5D questionnaire is a visual analog scale (VAS) where the participant is asked to rate their current overall health on a scale from 0 to 100, with 100 representing the best health imaginable and 0 the worst. People completing the EQ-5D are asked to do so base on their health on the same day.

### Inclusion criteria and data extracted

The study was conducted on adult participants of the RUDY Study, who had XLH and reported being caregivers of a relative with XLH and who completed the CarGOQoL by June 30, 2024. The RUDY Study has been recruiting people with rare musculoskeletal diseases, including XLH, since 2014. Participants are invited to complete all relevant questionnaires for their disease when they sign up and are reminded via text message every 6 mo to visit the platform again to complete follow-up or new questionnaires. A new question asking RUDY participants with XLH, “Do you provide any care for individual(s) with a rare disease?” was included in February of 2023. Those who answered “Yes” were asked how many people they cared for, whether they were adult or child, their rare disease, and then they were presented with the CarGOQoL questionnaire for them to complete. Participants with XLH self-declaring to be caregivers and who completed the CarGOQoL were included in the study, and those who answered “No” to the question about providing care were used as potential comparator non-caregivers. Crucially, as RUDY participants, caregivers were, by definition, people with XLH. Matching participants with XLH but who did not report being caregivers were included to conduct the comparison between caregivers and matched non-caregivers in the generic quality of life EQ-5D, as they would not have been asked to complete the caregiving-related CarGOQoL.

For each caregiver participant included in the study, we extracted their responses to the CarGOQoL questionnaire, whether they looked after a child or an adult, and how many people they looked after. For both caregivers and matched non-caregivers, gender, the EQ-5D questionnaire, age at time of data extraction, ethnicity, index of multiple deprivation derived from residential postcode, region of residence, whether they had XLH symptoms at birth, and other symptoms and co-morbidities were extracted.

### Type of study and design

This was a human observational cross-sectional study used to characterize the quality of life of caregivers of people with XLH, using primarily a caregiver-specific questionnaire completed only by caregivers. The study also compares the quality of life of caregivers to that of matched XLH participants who did not indicate being caregivers using the EQ-5D, which both groups completed.

### Statistical analysis

For the characterization of the quality of life of caregivers using the CarGOQoL and EQ-5D descriptive questionnaires, the number and proportion of responses by level for each item are presented, as well as the mean, median, and interquartile range (IQR) for the domain and CarGOQoL total score. For the EQ-5D, dimension responses are contrasted against those of the UK general population.^13^ For the EQ-5D VAS, mean, median, and IQR are reported. This analysis was conducted only on caregivers as non-caregivers did not complete the CarGOQoL.

To examine the association between the CarGOQoL and EQ-5D instruments, univariate linear regression models were fitted with CarGOQoL overall score as response variable and EQ-5D index, age, and gender as independent variables. If either age or sex were found to be significant (*p* < .05), a multivariate regression model was fitted including EQ-5D index and the significant predictor(s). Further univariate linear regression models were fitted with each of the EQ-5D dimensions (treated as categorical variables) as independent variables. Assumptions for linear regression were checked (details of the steps followed are provided in the Supplementary documentation) and diagnostics reported. Again, this analysis was also conducted only on caregivers, as non-caregivers did not complete the CarGOQoL.

Finally, to compare the EQ-5D findings between caregivers and non-caregivers, for each of the former we identified a non-caregiver (adults with XLH who completed the question about whether they provided any care for people with a rare disease and answered “No”) of the same gender and closest age and chose them as a matched reference. We describe the 2 groups in terms of all available participant characteristics and compare those using relevant statistical tests depending on the nature of variables. The Fisher’s exact test was used for categorical variables, Chi-square if the sample size per cell was greater than 5 and for binary variables, and the Mann–Whitney *U* Test for continuous variables. We then report the EQ-5D findings of the matched non-caregivers and compare them to those reported prior for the caregivers.

All analyses were conducted using R version 4.4.1 (2024-06-14 ucrt).^14^

## Results

At the time of data extraction from the RUDY Study, there were 187 participants in the dataset with XLH; 168 (89.8%) were adults and 72 of them (42.9%) completed the question about whether they provided any care for people with a rare disease. Of these, 16 (22.2%) answered “Yes” and 13 of those (81.2%) completed the CarGOQoL. All 13 caregivers reported caring for people with XLH, which suggests they were likely family members, and only 1/13 reported being paid. Table 1 shows the characteristics of the caregivers included in the study. They were mostly women (12/13) aged around 40, nearly half of English/Welsh/Scottish/Northern Irish/British ethnicity, from most regions of the United Kingdom, and less deprived than the general population. Nearly half of caregivers looked after an adult and the other half after a child, with the great majority being caregivers for 1 or 2 people. Abdominal pain was a common symptom reported by participants (6/13).

**Table 1:** Characteristics of participant caregivers included in the study.

**Variable**	**Caregivers (*n* = 13)**	
	***n***	**%**
**Gender**		
**Female**	12	92.31
**Male**	1	7.69
**Age (years)**		
**Median (IQR)**	44.92 (12)	
**Ethnicity**		
**English/Welsh/Scottish/Northern Irish/British**	6	46.15
**Other**	2	15.38
**Missing**	5	38.46
**Index of multiple deprivation**		
**Quintile 1 (most deprived)**	1	7.69
**Quintile 2**	0	0.00
**Quintile 3**	6	46.15
**Quintile 4**	3	23.08
**Quintile 5 (least deprived)**	2	15.38
**Missing**	1	7.69
**Region**		
**East Midlands**	1	7.69
**East of England**	1	7.69
**London**	2	15.38
**North West**	0	0.00
**South East**	2	15.38
**South West**	2	15.38
**West Midlands**	1	7.69
**Yorkshire and The Humber**	0	0.00
**Wales**	1	7.69
**Channel Islands**	1	7.69
**First symptom at date of birth**		
**Yes**	6	46.15
**Looked after a child or adult?**		
**Adult**	6	46.15
**Child**	6	46.15
**Adult and child**	1	7.69
**Number of persons the caregiver looked after**		
**1**	8	61.54
**2**	4	30.77
**3**	1	7.69
**Symptoms and co-morbidities (most common)**		
**Abdominal pain**	6	46.15
**Abnormal heart**	2	15.38
**Diabetes**	2	15.38
**Count of symptoms and co-morbidities (per patient)**		
**0-5**	2	15.38
**6-10**	3	23.08
**11-15**	4	30.77
**16-20**	2	15.38
**21+**	2	15.38
**Mean**	14.62	
**Median (IQR)**	11 (9)	

Abbreviation: IQR, interquartile range.

### Characterization of quality of life of caregivers

**Figure 1:**
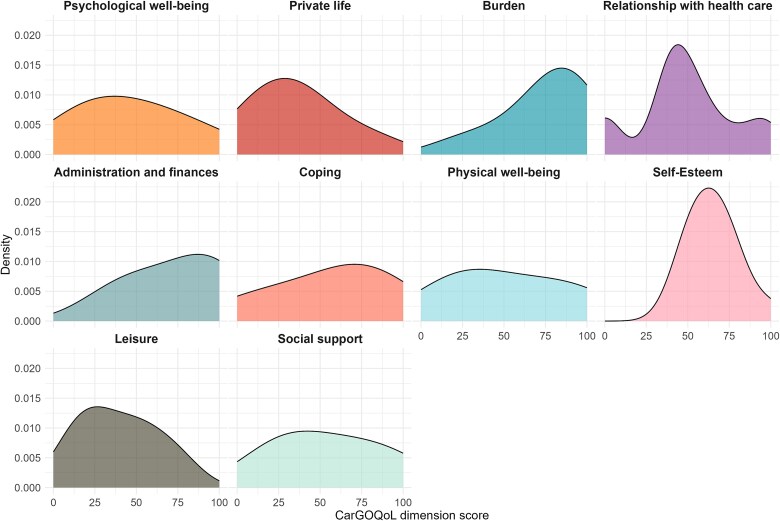
Density of CarGOQoL dimension scores by caregivers. This figure shows density plots illustrating the distribution of CarGOQoL domain scores calculated from responses to the corresponding questions by RUDY participants reporting to be caregivers. The x-axis represents the domain score, where a higher score indicates better quality of life, while the y-axis shows the density estimate. The plots were generated using a kernel density estimation method, with peaks indicating domain scores more commonly reported. Abbreviations: CarGOQoL, caregiver oncology quality of life questionnaire; RUDY, Rare UK Diseases Study.

**Figure 2:**
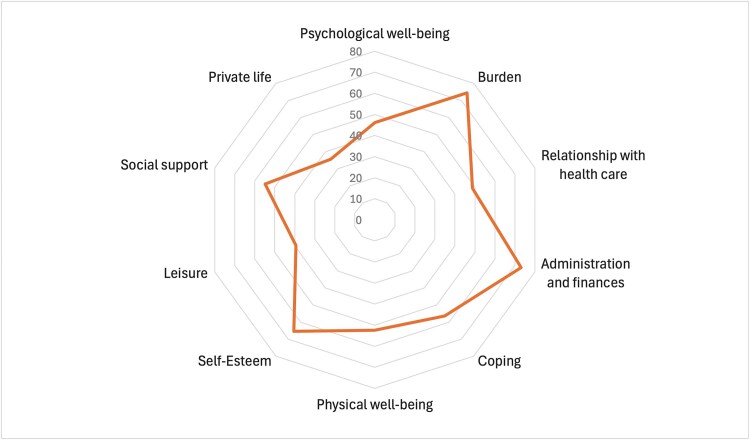
Mean CarGOQoL domain scores by caregivers. This spider diagram displays the mean score for each of the 10 CarGOQoL domains calculated from responses to the corresponding questions by RUDY participants reporting to be caregivers. Each axis represents 1 of the domains, with possible values ranging between 0 and 100 (shown up to 80 because no mean score was above 80). Higher scores indicate better quality of life, with the area inside the orange line representing the overall mean quality of life Vis-à-Vis the maximum possible quality of life represented by the outermost ring. Abbreviations: CarGOQoL, caregiver oncology quality of life questionnaire; RUDY, Rare UK Diseases Study.

Table 2 shows the responses by XLH caregivers to the 29 items of the CarGOQoL questionnaire. After applying the item reversion to the corresponding questions, scores by domain were generated for each caregiver and their density for the full sample is shown in Figure 1. The distribution of caregivers’ responses over the various domains varied greatly, with *burden* for example reporting a clearly left-skewed distribution with most individual scores toward the high end (and better quality of life) (median = 81), whereas for *social support* the distribution shows reduced variability of scores throughout the entire range of possible values (median = 50). Figure 2 illustrates the mean domain scores, showing those where caregivers report better quality of life (*burden*, *administration and finance*, and *self-esteem*), and those where they do worst (*private life*, *leisure*, and *psychological wellbeing*). Summary statistics for CarGOQoL domain scores for caregivers are reported in the Table S2 of the Supplementary documentation. The mean overall CarGOQoL score was 54.65. A histogram and density plot of the distribution of CarGOQoL overall scores for XLH caregivers is provided in Figure S1 of the Supplementary documentation.

**Table 2:** Responses to items in CarGOQoL questionnaire.

**Domain/item**	**Never/** **not at all, *n* (%)**	**Rarely/** **a little, *n* (%)**	**Sometimes/** **moderately, *n* (%)**	**Often/** **a lot, *n* (%)**	**Always/** **enormously, *n* (%)**
**Psychological well-being**					
**Been worried, anxious?**	2 (15.38)	2 (15.38)	3 (23.08)	5 (38.46)	1 (7.69)
**Been sad, depressed?**	3 (23.08)	4 (30.77)	1 (7.69)	4 (30.77)	1 (7.69)
**Been emotionally tired, worn out?**	1 (7.69)	2 (15.38)	4 (30.77)	2 (15.38)	4 (30.77)
**Been stressed?**	2 (15.38)	0 (0.00)	4 (30.77)	5 (38.36)	2 (15.38)
**Burden**					
**Felt a lack of freedom?**	2 (15.38)	6 (46.15)	2 (15.38)	2 (15.38)	1 (7.69)
**Been bothered by the feeling of being confined?**	5 (38.46)	3 (23.08)	2 (15.38)	2 (15.38)	1 (7.69)
**Been bothered by the fact that your life was entirely devoted to the care recipient?**	8 (61.54)	3 (23.08)	0 (0.00)	1 (7.69)	1 (7.69)
**Been embarrassed to be the only person to provide assistance?**	9 (69.23)	2 (15.38)	2 (15.38)	0 (0.00)	0 (0.00)
**Relationship with health care**					
**Been satisfied with information given by health care providers (doctors, nurses)?**	2 (15.38)	1 (7.69)	4 (30.77)	3 (23.08)	3 (23.08)
**Been reassured by the health care providers (doctors, nurses)?**	2 (15.38)	2 (15.38)	4 (30.77)	3 (23.08)	2 (15.38)
**Felt that your role as caregiver was recognized by health care providers (doctors, nurses)?**	4 (30.77)	4 (30.77)	1 (7.69)	3 (23.08)	1 (7.69)
**Administration and finances**					
**Had financial difficulties (lodging, transportation)?**	5 (38.46)	3 (23.08)	4 (30.77)	1 (7.69)	0 (0.00)
**Had other difficulties (lodging, transportation)?**	5 (38.46)	3 (23.08)	3 (23.08)	2 (15.38)	0 (0.00)
**Encountered difficulties in the administrative process (health insurance paperwork and other paperwork related to the cancer illness?)**	7 (53.85)	2 (15.38)	1 (7.69)	3 (23.08)	0 (0.00)
**Coping**					
**Experienced feelings of guilt?**	4 (30.77)	3 (23.08)	1 (7.69)	3 (23.08)	2 (15.38)
**Been bothered by a feeling of helplessness against disease?**	2 (15.38)	2 (15.38)	3 (23.08)	4 (30.77)	2 (15.38)
**Felt a feeling of injustice, anger, or rebellion?**	5 (38.46)	3 (23.08)	2 (15.38)	1 (7.69)	2 (15.38)
**Physical wellbeing**					
**Had sleeping difficulties?**	3 (23.08)	3 (23.08)	1 (7.69)	4 (30.77)	2 (15.38)
**Had problems with your appetite?**	7 (53.85)	2 (15.38)	3 (23.08)	1 (7.69)	0 (0.00)
**Been physically tired, worn out?**	2 (15.38)	1 (7.69)	3 (23.08)	1 (7.69)	6 (46.15)
**Had the impression that your health was fragile?**	4 (30.77)	0 (0.00)	2 (15.38)	3 (23.08)	4 (30.77)
**Self-esteem**					
**Felt you made a difference for the person you are helping?**	0 (0.00)	0 (0.00)	5 (38.46)	7 (53.85)	1 (7.69)
**Felt useful?**	0 (0.00)	1 (7.69)	5 (38.46)	6 (46.15)	1 (7.69%)
**Leisure**					
**Could rest, relax?**	3 (23.08)	5 (38.46)	2 (15.38)	3 (23.08)	0 (0.00)
**Could take care of yourself, pay attention to your own health?**	0 (0.00)	6 (46.15)	4 (30.77)	3 (23.08)	0 (0.00)
**Social support**					
**Been assisted, supported, understood by your family?**	1 (7.69)	2 (15.38)	5 (38.46)	1 (7.69)	4 (30.77)
**Been assisted, supported, understood by your friends?**	2 (15.38)	3 (23.08)	3 (23.08)	3 (23.08)	2 (15.38)
**Private Life**					
**Had difficulties in your intimate, emotional life?**	1 (7.69)	2 (15.38)	2 (15.38)	6 (46.15)	2 (15.38)
**Had a satisfying love and sexual life?**	3 (23.08)	5 (38.46)	3 (23.08)	2 (15.38)	0 (0.00)

Abbreviation: CarGOQoL, Caregiver Oncology Quality of Life Questionnaire.

**Table 3:** Summary statistics of EQ-5D for caregivers.

	**No problem, *n* (%)**	**Slight problems, *n* (%)**	**Moderate problems, *n* (%)**	**Severe problems, *n* (%)**	**Extreme problems/unable, *n* (%)**
**Dimension**					
**Mobility**	3 (23.08)	3 (23.08)	4 (30.77)	2 (15.38)	1 (7.69)
**Self-care**	7 (53.85)	1 (7.69)	3 (23.08)	2 (15.38)	0 (0.00)
**Usual activities**	3 (23.08)	4 (30.77)	4 (30.77)	2 (15.38)	0 (0.00)
**Pain/Discomfort**	3 (23.08)	3 (23.08)	3 (23.08)	2 (15.38)	2 (15.38)
**Anxiety/Depression**	4 (30.77)	1 (7.69)	4 (30.77)	3 (23.08)	1 (7.69)

**Figure 3:**
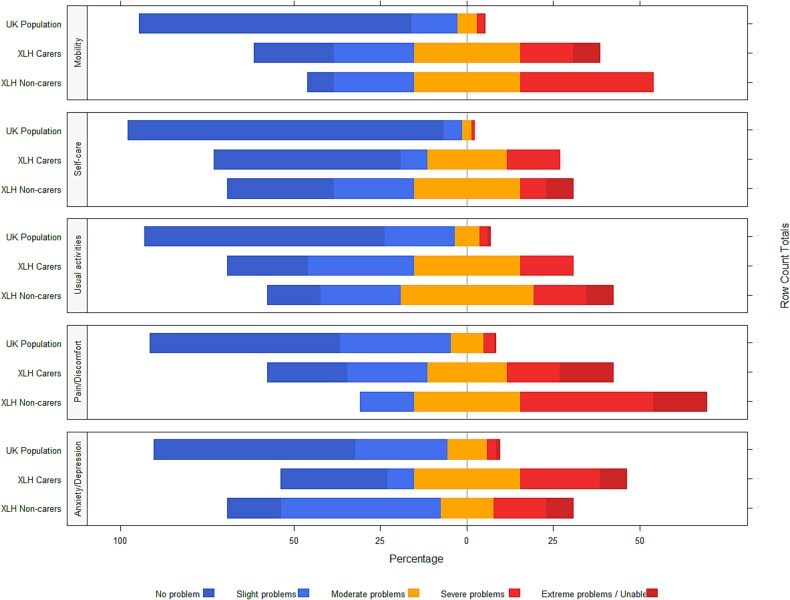
Responses to EQ-5D questionnaire by caregivers, matched non-caregivers, and the general population. This horizontal stacked bar chart illustrates the distribution of responses from caregivers, matched non-caregivers, and the general population on the 5 response levels of the EQ-5D, ranging from no problems to extreme problems. A set of 3 stacked bars (1 per group) is presented for each of the 5 EQ-5D dimensions. Stacks indicate the proportion of responses for each response level, with bars centered so that the middle point of the middle stacks (“moderate problems”) align across all groups. The x-axis shows the percentage of responses worse than the middle point (to the right) and better than the middle point (to the left). The figure highlights the differences in response distributions among the groups, with larger bars to the right indicating higher proportions of responses worse than the middle point of “moderate problems.” Data for XLH caregivers and matched non-caregivers were collected from the RUDY study, while those of the general population are taken from a previously published study. Abbreviations: RUDY, Rare UK Diseases Study; XLH, X-linked hypophosphatemia.

**Figure 4:**
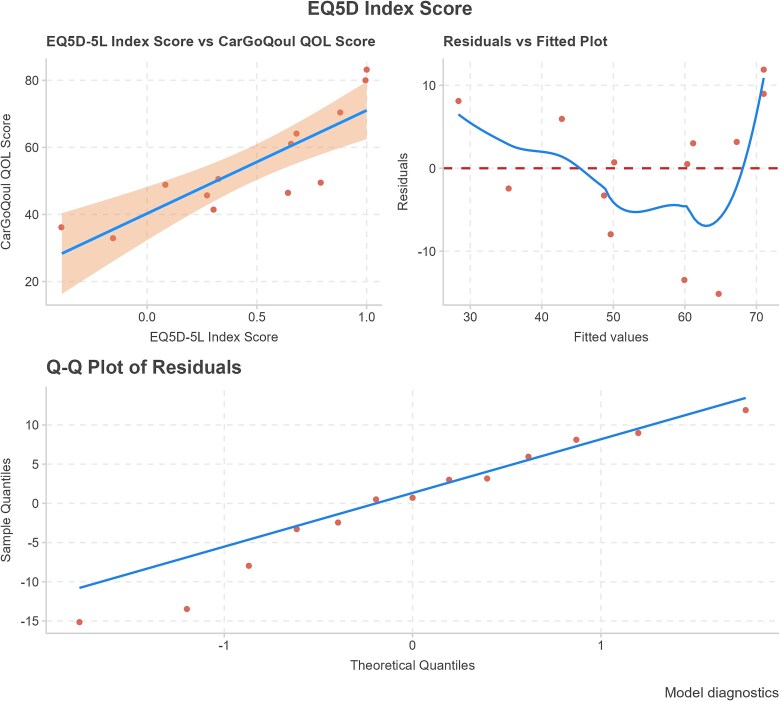
Univariate CarGOQoL = EQ-5D regression model performance. The top-left plot shows the linear relationship between EQ-5D score and CarGOQoL overall score. The line represents the regression fit, and the shaded region indicates 95% confidence intervals. The top-right plot displays residuals versus fitted values. This plot provides information regarding the quality of the regression model. Ideally, a well-fitted model should show residuals randomly scattered around zero, with no apparent patterns. The bottom plot is a Q-Q plot, which checks if residuals follow a normal distribution. Significant deviations from the diagonal line indicate potential non-normality.

Results by EQ-5D domain are shown in Table 3, and in Figure 3 next to those of the UK general population for context. Caregivers do most noticeably poorly on mobility, pain/discomfort, and anxiety/depression, and they report more problems in all dimensions compared to the general population. Mean EQ-5D score was 0.467, with 2 caregivers reporting no problem in all 5 dimensions (the maximum EQ-5D score of 1) and the lowest being −0.388 with no 1 indicating extreme problems in all five. Median score was 0.639 and IQR 0.521. Caregivers reported a mean VAS score of 55.85, the lowest being 18, the maximum 90, and median and IQR 60 and 50, respectively.

### Association between CarGOQoL and EQ-5D

Model results and diagnostics for all univariate regression models are reported in Table S3 of the Supplementary documentation. Age and gender were not statistically significant predictors of CarGOQoL overall score so no further multivariate model was fitted to examine the relationship between EQ-5D and CarGOQoL. The univariate regression with the EQ-5D score tested if was significantly associated with the CarGOQoL overall score. The resulting model was CarGOQoL overall score = 40.280 + 30.749*(EQ-5D score). A significant association was found between EQ-5D score and CarGOQoL overall score (*p* < .001); however, inspection of model diagnostics (Figure 4) suggests potential deviations from normality in the residuals based on the Q-Q plot of residuals. Additionally, the residual-versus-fitted plot shows extreme residuals, especially toward the high end of fitted values, indicating possible heteroscedasticity. Regression models with each EQ-5D dimension as independent variables were statistically significant for Self-care, Usual activities, and Pain/discomfort but not for Mobility or Anxiety/Depression (see Supplementary documentation, Figure S2 for details).

### Generic quality of life of caregivers vs. non-caregivers

All caregivers were successfully matched to another RUDY participant with XLH who declared not being a caregiver. Characteristics of each group and results of statistical tests assessing whether they were statistically different are reported in Table S4 of the Supplementary documentation. Matching on closest age led to nearly equal mean ages between the groups (44.9 yr for caregivers and 44.5 yr for non-caregivers). Although there were no statistically significant differences between any of the variables for which data on their characteristics were available between the groups, a much larger proportion of matched non-caregivers were English/Welsh/Scottish/Northern Irish/British (11/13 vs 6/13 for caregivers). A larger proportion of caregivers also reported XLH symptoms at birth (6/13 vs 2/13 for matched non-caregivers), with the mean number of other symptoms and co-morbidities being higher as well for caregivers (14.6 vs 12.3 for matched non-caregivers). Notably, while 6/13 of caregivers reported abdominal pain, only 2/13 of matched non-caregivers did.

Generic quality of life was quite different between groups. Figure 3 shows dimension-level responses for caregivers, matched non-caregivers, and the UK general population for reference. The figure shows how matched non-caregivers reported worse quality of life than caregivers in all EQ-5D dimensions except for Anxiety/Depression. Specific values for matched non-caregivers are reported in Table S5 of the Supplementary documentation. The Pain/discomfort dimension stands out: while 3.5% of the general population report severe or extreme pain or discomfort,^13^ 4/13 (30.8%) of caregivers in our study did, and for matched non-caregivers, the figure was 53.8% (7/13).

The EQ-5D score and VAS reflect the findings at the dimension level. Matched non-caregivers reported worse quality of life, with a mean EQ-5D score of 0.356 (0.112 or 24% lower than that of caregivers), median of 0.426, a minimum reported score of −0.245, and a maximum of 0.837, indicating that none of the matched non-caregivers reported no problem in all dimensions. IQR was 0.242, less than half of that found for caregivers, pointing to scores for matched non-caregivers being more concentrated around the median than for caregivers. The mean for the VAS was 53.15 (8 points and 13% lower than reported by caregivers), with a median of 54, values ranging from 15 to 95, and IQR of 20.

## Discussion

We conducted a detailed analysis of the quality of life of people with XLH who are caregivers of relatives with XLH. We found that dealing with the administration and finance associated with the disease does not impact them negatively, and that, in general, the caregiving is not felt as a burden and neither does it impact their self-esteem. However, it does limit their private life, their ability to engage in leisure activities, and ultimately, they pay a price in terms of their psychological wellbeing, ie, feeling worried, depressed, sad, worn out, or stressed. This is largely consistent with other studies reporting on the experience of caregivers of people with rare diseases. A recently published review about the experiences of quality of life and access to health services among rare disease caregivers found 13 studies reporting evidence linking caring for people with rare diseases to lower scores in the psychological dimension of quality of life, including anxiety and depression.^15^ But the positive impact of caregiving, which in our study is captured by the fact that despite the negative consequences, caregivers do not feel it is a burden, has also been reported in the literature with evidence of both psychological challenges as well as gratification and a sense of purpose.^16^

Another study which used the CarGOQoL but to assess the quality of life of caregivers of people with cancer in 3 different sites (digestive, lung, and breast) found administration and finance, burden, and self-esteem to be the least impacted areas of quality of life, with psychological wellbeing and leisure being the most.^17^ The scores by dimension identified for the 3 cancers examined are, except one, quite similar to those we found in our analysis. The dimension in which scores were most different was private life, which was rated much worse in our study than for the caregivers of people with cancer (35.6 for caregivers of people with XLH vs 75.8 on average for caregivers of people with cancer). This can likely be explained by the fact that the caregivers of people with XLH in our study also have XLH, which is expected to affect their private lives significantly. In all 10 dimensions, however, caregivers of people with XLH reported mean scores lower (worse than) caregivers of people with all 3 types of cancer.

We found that, in terms of their more generic quality of life, caregivers of people with XLH report more problems in mobility, self-care, carrying out usual activities, pain or discomfort, and anxiety or depression compared to the general UK population. This is to be expected considering that not only are they caregivers and performing that role will come with a negative impact on their quality of life as described above, but they also have XLH, which directly impacts quality of life. Clinical signs and symptoms of XLH in adults are known to be dominated by pain but can also include skeletal pathology (rickets, short stature, lower limb deformity, spinal stenosis, dental anomalies, enthesopathies, hearing loss), stiffness and muscle function deficits (in lower limb power and functional capacity), generalized impaired mobility and physical function, joint issues and surgeries, and other physical problems.^18^^,^^19^

Our statistical analysis showed that the EQ-5D score was associated with the CarGOQoL overall score, although some inconsistencies found in the residuals suggest this association should be taken with caution. That both scores move in the same direction is expected and reassuring as they both aim to measure quality of life. The large residuals suggest that a perfect prediction is not possible, which is expected as the 29 items of the caregiver-specific CarGOQoL capture the state of the respondent in dimensions of quality of life that are absent in the EQ-5D. These findings are common in the literature when assessing individuals with both a generic and a specific quality of life questionnaire, as was found in a study of patients with COPD who completed the EQ-5D and 2 disease-specific questionnaires.^20^ However, the use of standard methods such as the EQ-5D to evaluate health-related quality of life and disease burden for people with rare diseases and their caregivers has been found to lead to significant uncertainties in submissions to health technology assessment bodies such as the UK’s NICE. A review of 24 NICE Highly Specialized Technology submissions found that the EQ-5D may not capture the full impact of rare diseases due to its limited domains,^21^ which is supported by our findings from examining the association between the detailed CarGOQoL and the more limited and generic EQ-5D. The authors also found that using single-arm studies has led to bias in results, and that the methods commonly used in submissions (including using proxy data, limited duration of application, and low number of caregivers) are often criticized.^21^

Following from this and our findings, using both the CarGOQoL and the EQ-5D not as a substitute for each other but as complementary questionnaires can provide a more sensitive and comprehensive picture of the impact of caregiving. The CarGOQoL can provide the more sensitive measurement relating to the impact of caregiving, while the EQ-5D is limited to generic quality of life but benefits from being a preference-based instrument which can be used to estimate health utility, often needed for economic evaluations that inform decision-making in health. It is nonetheless important to recognize that new methodologies are still needed to better reflect the impact of rare diseases on people who have the disease and especially those who are caregivers. More flexible guidance and acceptance of alternative health-related quality of life measures as well as clear recommendations on the estimation of utilities across different rare diseases, including the impact of bereavement, are needed.^21^

The fact that most caregivers were women deserves a special note as the implications are multifaceted and significant. This carries as economic impact as women caregivers may have to reduce their working hours or leave their jobs entirely to provide care, leading to financial strain and loss of income.^22^ In turn, this can result in long-term economic disadvantages, including reduced retirement savings and increased poverty risk. Female caregivers often experience higher levels of stress, depression, and physical health issues compared to their male counterparts.^22^^,^^23^ The emotional and physical burden of caregiving can lead to burnout and negatively impact their overall well-being, including limiting social interactions and opportunities for personal development, leading to feelings of isolation and loneliness. This predominance of women as unpaid caregivers reinforces traditional gender roles and contributes to gender inequality in both the home and workplace, risks to perpetuate stereotypes and limits women’s opportunities for career advancement and personal growth.

Lastly, we found that caregivers reported better quality of life than their matched non-caregivers from RUDY in all EQ-5D dimensions except for Anxiety/Depression. This finding points directly at the hypothesis that in families of more than 1 person with XLH, the 1 with better quality of life will be the 1 taking on the role of caregiver. Previous studies have highlighted this, where after controlling for demographic variables, healthier individuals were found to be much more likely to be caregivers.^24^ The help and support provided by caregivers requires that they are relatively mobile, unquestionably able to look after themselves so that they could look after someone else, and able to perform usual activities. These were all reflected in our findings, with caregivers reporting less severe problems in those 3 dimensions of the EQ-5D than their matched non-caregivers. An association between pain and disease severity for people with XLH is to be expected, as has been identified for other musculoskeletal diseases.^25^ So again, our finding that caregivers reported less problems in pain/discomfort than their matched non-caregivers suggests they may be experiencing a less severe presentation of the disease, and 1 that would allow them to serve as caregivers. But this pattern was broken when assessing anxiety and depression via the EQ-5D, with caregivers reporting more problems likely due to their caring role, which as described earlier comes with the undesirable consequence of important limitations to private life, leisure, and overall impact on their psychological wellbeing. However, it is important to recognize that, alongside the negative impacts of caregiving, there are also benefits from caregiving which may contribute to caregivers reporting better quality of life. Caregivers often experience increased confidence, improved problem-solving skills, a stronger bond with the care recipient, and reassurance that the care recipient is receiving proper care,^26^ all of which may contribute to an improved quality of life. The association between better quality of life and being a caregiver may hence come from both directions, ie, better quality of life could be the enabler of caregiving, the consequence, or both. Future research should examine these potential causal pathways to better understand the impact of caregiving on the quality of life of the caregivers, especially in the context of rare diseases.

Caregivers of people with XLH play an important role in supporting the everyday life of those they look after. This role should be recognized, and helpful information and tools made available to support them in that process. Understanding caregivers as strong, resilient, able, and competent partners is an important step.^27^ They should be supported, including by identifying secondary caregivers especially in the case of XLH as they take on the consequences of living with the disease and supporting family members. Further research to better understand the distinction between the quality of life impact of the caring activity and that from the natural consequences of the disease should be conducted. Subsequent similar studies are also warranted to capture the effect that potential new therapies or health management pathways may have on caregiving status and burden.

This study benefited from the direct involvement of volunteer XLH caregivers who helped in the selection of the CarGOQoL as the most appropriate instrument to capture the impact of being a caregiver of someone with the disease. We were able to use observational data provided directly by participants from all over the United Kingdom, which included personal characteristics allowing us to describe our groups in detail and to match caregivers to non-caregivers based on gender and age. There were also important limitations, such as the sample size, which is generally a challenge for studies of rare diseases. By using RUDY we captured information from a less deprived, more informed, and proactive patient population. The selection of caregivers was based on their self-identification as “caregivers,” which possibly led to people who do provide care and support to family members with the disease to be regarded as non-caregivers simply because they did not identify themselves with such term. Self-identification as a caregiver is considered to be a socially constructed process often characterized by a delay between undertaking caregiving tasks and recognizing oneself as a caregiver.^28^ This means that some people reporting not to be caregivers actually might have been. If that was the case on our study, our results would have been biased making the differences we report between caregivers and matched non-caregivers to appear larger than they actually are. However, to be classed as “caregivers” on our study, RUDY participants were presented with a question not about whether they were “caregivers” but about whether they provided “any care for individual(s) with a rare disease?”, which we expect would have helped to mitigate this potential problem. People who reported to provide care also might actually not do so, which would introduce bias in the opposite direction, but we consider this less likely especially in the context of debilitating rare diseases. Another potential source of bias is that we could not control for the number of family members with XLH in the matched non-caregiver group. Unfortunately, this information was not available in the RUDY dataset. The platform does not ask participants how many people in their family have XLH, nor does it link participants who are members of the same family. Having access to this information would allow for a better matching and more accurate measures of the impact of caregiving. Finally, and as highlighted above, the EQ-5D is likely unable to capture all the health-related quality of life relevant aspects for people with XLH and especially those who are caregivers, and this calls for the inclusion of other instruments such as the CarGOQoL which, although not intended to measure quality of life in general, it does provide a more thorough picture of the impact of caregiving on their quality of life of those who provide care.

This study characterized the quality of life of caregivers of people with XLH and found caregiver-specific and generic quality of life scores to be consistently correlated. Comparing their quality of life to that of matched non-caregivers adds to the evidence that identifies caregivers as healthier, able, and resilient partners who assume psychological challenges in the process.

## Supplementary Material

Supplementary_documentation_final_ziaf078

## Data Availability

The data underlying this article cannot be shared publicly due to the privacy of individuals that participated in the RUDY Study. The data can be shared upon reasonable request to the corresponding author following approval from the RUDY Study Data Access Committee (https://rudystudy.org/).
