# Supplementary material for: Double burdened yet resilient: quality of life of caregivers of people with X-linked hypophosphatemia
Source: JBMR Plus. 2025 Apr 30;9(7):ziaf078. doi: 10.1093/jbmrpl/ziaf078 (PMC12143474; doi:10.1093/jbmrpl/ziaf078)
Supplement: Supplementary_documentation_final_ziaf078 [file supplementary_documentation_final_ziaf078.docx]

**Double burdened yet resilient: quality of life of caregivers of people with x-linked hypophosphatemia**

Supplementary documentation

**Table S1. CarGOQoL domains, items and item reversion for scoring purposes (do not use without permission).**

| Domain | Item | Item reversion |
| --- | --- | --- |
| Psychological well-being | Been worried, anxious? | Yes |
|  | Been sad, depressed? | Yes |
|  | Been emotionally tired, worn out? | Yes |
|  | Been stressed? | Yes |
| Burden | Felt a lack of freedom? | Yes |
|  | Been bothered by the feeling of being confined? | Yes |
|  | Been bothered by the fact that your life was entirely devoted to the care recipient? | Yes |
|  | Been embarrassed to be the only person to provide assistance? | Yes |
| Relationship with  health care | Been satisfied with information given by health care providers (doctors, nurses…)? | No |
|  | Been reassured by the health care providers (doctors, nurses…)? | No |
|  | Felt that your role as caregiver was recognized by health care providers (doctors, nurses…)? | No |
| Administration  and finances | Had financial difficulties (lodging, transportation…)? | Yes |
|  | Had other difficulties (lodging, transportation…)? | Yes |
|  | Encountered difficulties in the administrative process (health insurance paperwork and other paperwork related to the cancer illness)? * | Yes |
| Coping | Experienced feelings of guilt? | Yes |
|  | Been bothered by a feeling of helplessness against disease? | Yes |
|  | Felt a feeling of injustice, anger, or rebellion? | Yes |
| Physical well-  being | Had sleeping difficulties? | Yes |
|  | Had problems with your appetite? | Yes |
|  | Been physically tired, worn out? | Yes |
|  | Had the impression that your health was fragile? | Yes |
| Self-Esteem | Felt you made a difference for the person you are helping? | No |
|  | Felt useful? | No |
| Leisure | Could rest, relax? | No |
|  | Could take care of yourself, pay attention to your own health? | No |
| Social support | Been assisted, supported, understood by your family? | No |
|  | Been assisted, supported, understood by your friends? | No |
| Private Life | Had difficulties in your intimate, emotional life? | Yes |
|  | Had a satisfying love and sexual life? | No |

*: the reference to “cancer” on this question was replaced by “rare disease” to capture the difficulties encountered on paperwork related to XLH.

**Table S2. Summary statistics of CarGOQoL domain scores for caregivers**

| **Domain** | **Min** | **Mean** | **Median** | **IQR** | **Max** |
| --- | --- | --- | --- | --- | --- |
| Psychological well-being | 0.00 | 46.15 | 43.75 | 50.00 | 100.00 |
| Burden | 18.75 | 74.52 | 81.25 | 31.25 | 100.00 |
| Relationship with health care | 0.00 | 48.72 | 41.67 | 16.67 | 100.00 |
| Administration and finances | 25.00 | 73.08 | 75.00 | 50.00 | 100.00 |
| Coping | 0.00 | 56.41 | 66.67 | 41.67 | 100.00 |
| Physical well-being | 12.50 | 52.40 | 37.50 | 62.50 | 100.00 |
| Self-Esteem | 50.00 | 65.38 | 62.50 | 25.00 | 100.00 |
| Leisure | 12.50 | 39.42 | 37.50 | 25.00 | 75.00 |
| Social support | 12.50 | 54.81 | 50.00 | 50.00 | 100.00 |
| Private life | 0.00 | 35.58 | 37.50 | 37.50 | 87.50 |
| **Overall Score - QoL** | **32.92** | **54.65** | **49.58** | **18.75** | **82.92** |

**Checking for linear regression assumptions**

To assess the linearity between the response variable and each continuous covariate, scatter plots were generated, with categorical variables converted into numerical form to visualize linear relationships. The assumption of homoscedasticity (constant variance of residuals across levels of the independent variables) was evaluated by plotting the residuals against the fitted values. The assumption was satisfied if no discernible pattern appeared in this plot. To test the normality of the residuals, a Q-Q plot was used. The normality assumption was considered met if the points closely followed the diagonal reference line on the Q-Q plot.

**Figure S1 – Histogram and density plot of CarGOQoL overall score for XLH caregivers**


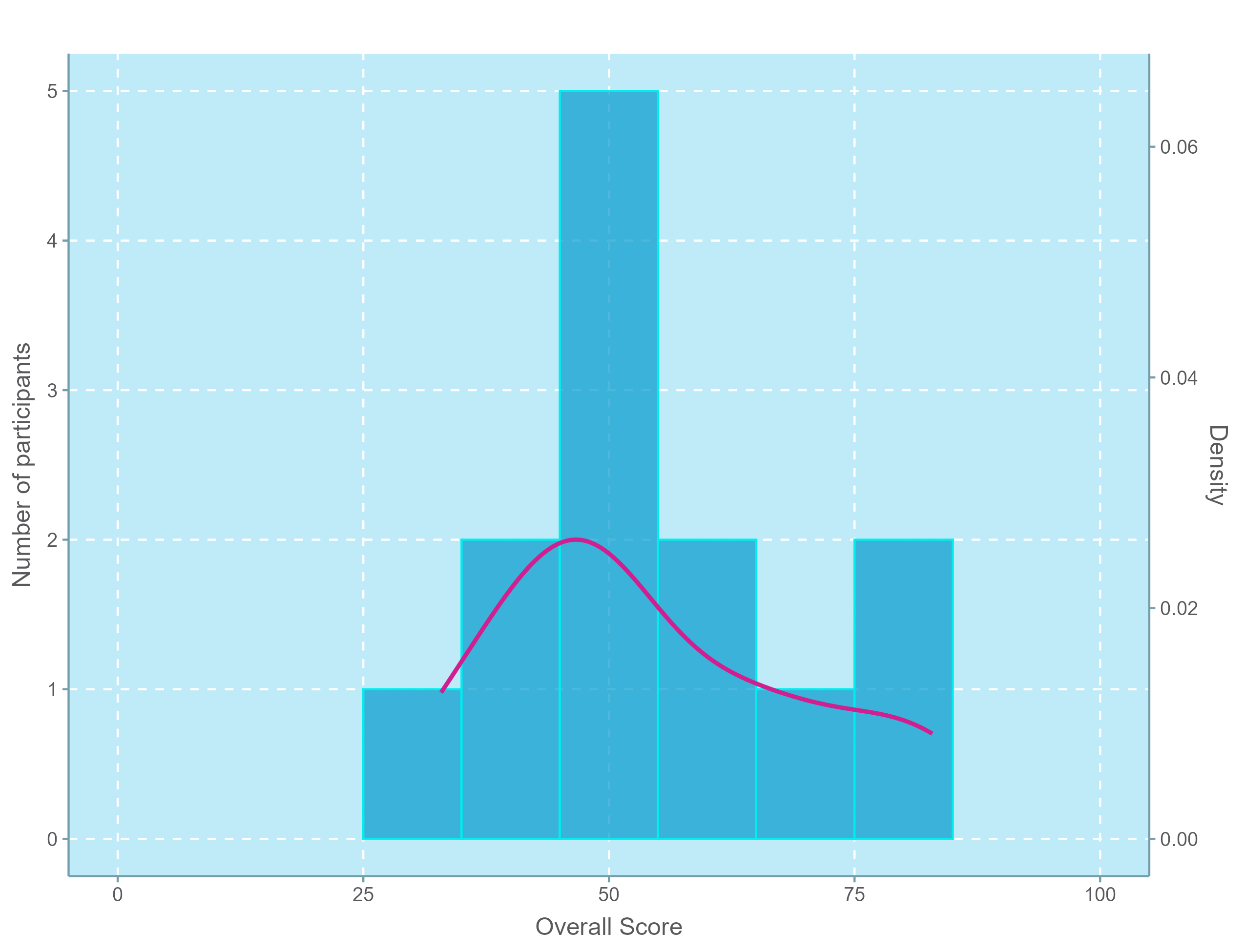


**Table S3. Results of univariate linear regression models with CarGOQoL overall score as response variable**

| **Predictor variable** | **Estimate (β)** | **Standard error** | **t-value** | **Pr(>\|t\|)** | **Adjusted  R-squared** |
| --- | --- | --- | --- | --- | --- |
| EQ-5D index | 30.749 | 5.727 | 5.369 | 0.000 *** | 0.699 |
| Age | -0.089 | 0.487 | -0.183 | 0.858 | -0.088 |
| Female gender | 27.465 | 15.122 | 1.816 | 0.097 | 0.161 |

Significance codes: 0 “***” 0.001 “**” 0.01 “*” 0.05 “.” 0.1 “ ” 1

| **Predictor variable** | **Estimate (β)** | **Standard error** | **t-value** | **Pr(>\|t\|)** | **Adjusted  R-squared** | **Significance F** |
| --- | --- | --- | --- | --- | --- | --- |
| **Mobility (Reference level = No problem)** | | | | | 0.376 | 0.100 |
| Slight problems | -10.486 | 10.233 | -1.025 | 0.335 |  |  |
| Moderate problems | -20.313 | 9.572 | -2.122 | 0.067 |  |  |
| Severe problems | -31.667 | 11.441 | -2.768 | 0.024 * |  |  |
| Unable | -34.375 | 14.472 | -2.375 | 0.045 * |  |  |
| **Self-care (Reference level = No problem)** | | | | | 0.467 | 0.034 |
| Slight problems | -14.077 | 12.376 | -1.137 | 0.285 |  |  |
| Moderate problems | -19.633 | 7.989 | -2.458 | 0.036 * |  |  |
| Severe problems | -30.223 | 9.282 | -3.256 | 0.010 ** |  |  |
| **Usual activities (Reference level = No problem)** | | | | | 0.841 | <0.001 |
| Slight problems | -22.517 | 4.831 | -4.661 | 0.001 *** |  |  |
| Moderate problems | -31.111 | 4.831 | -6.439 | 0.000 *** |  |  |
| Severe problems | -43.09 | 5.775 | -7.462 | 0.000 *** |  |  |
| **Pain / Discomfort (Reference level = No problem)** | | | | | 0.888 | <0.001 |
| Slight problems | -19.583 | 4.332 | -4.521 | 0.002 *** |  |  |
| Moderate problems | -29.097 | 4.332 | -6.717 | 0.000 *** |  |  |
| Severe problems | -34.236 | 4.843 | -7.069 | 0.000 *** |  |  |
| Extreme problems | -43.09 | 4.843 | -8.897 | 0.000 *** |  |  |
| **Anxiety / Depression (Reference level = No problem)** | | | | | 0.466 | 0.057 |
| Slight problems | -6.563 | 12.956 | -0.507 | 0.626 |  |  |
| Moderate problems | -22.135 | 8.194 | -2.702 | 0.027 * |  |  |
| Severe problems | -30.66 | 8.85 | -3.464 | 0.009 ** |  |  |

Significance codes: 0 “***” 0.001 “**” 0.01 “*” 0.05 “.” 0.1 “ ” 1

**Figure S2. Model diagnostics for univariate regressions with CarGOQoL overall score as response variable**


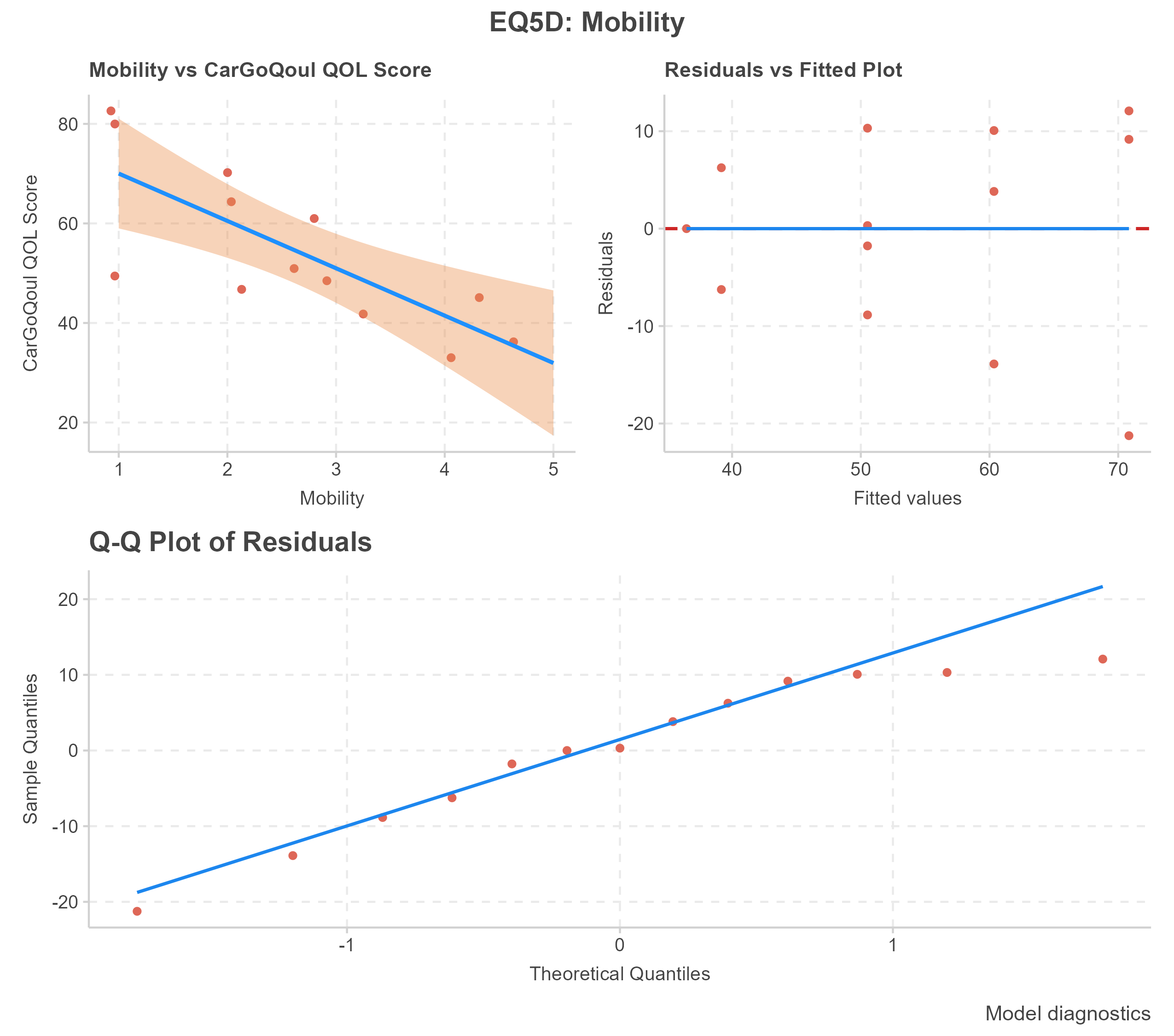


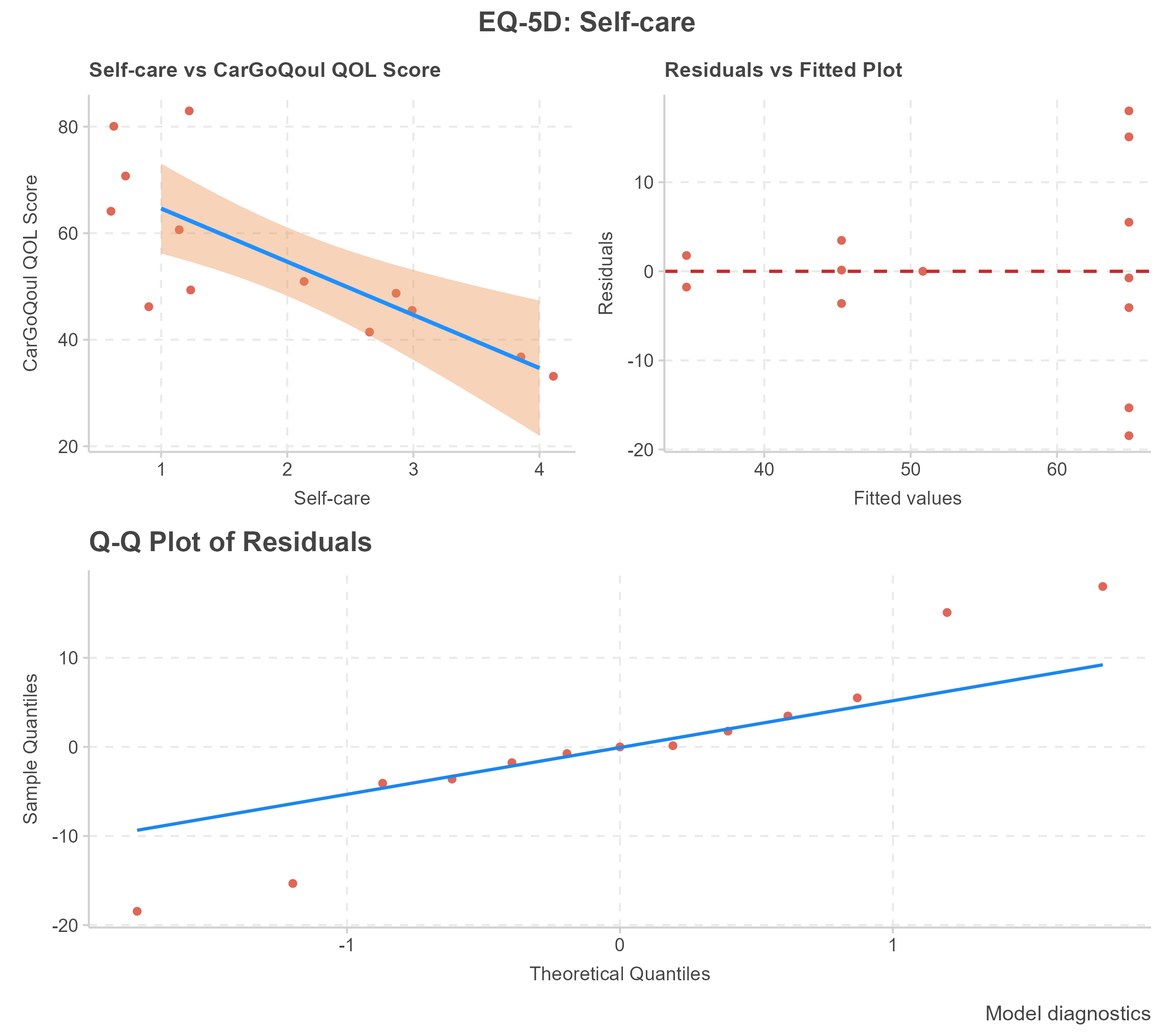


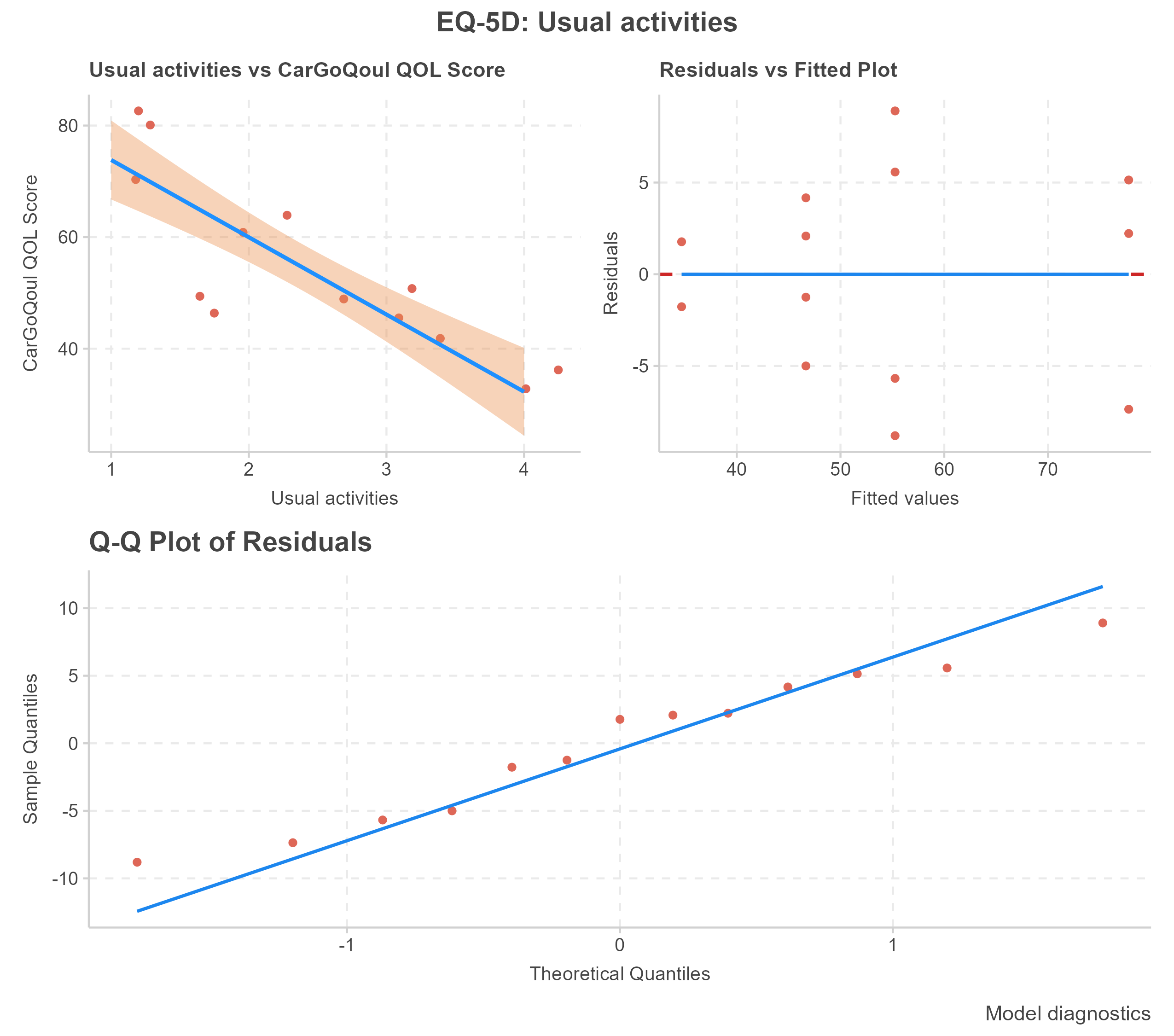


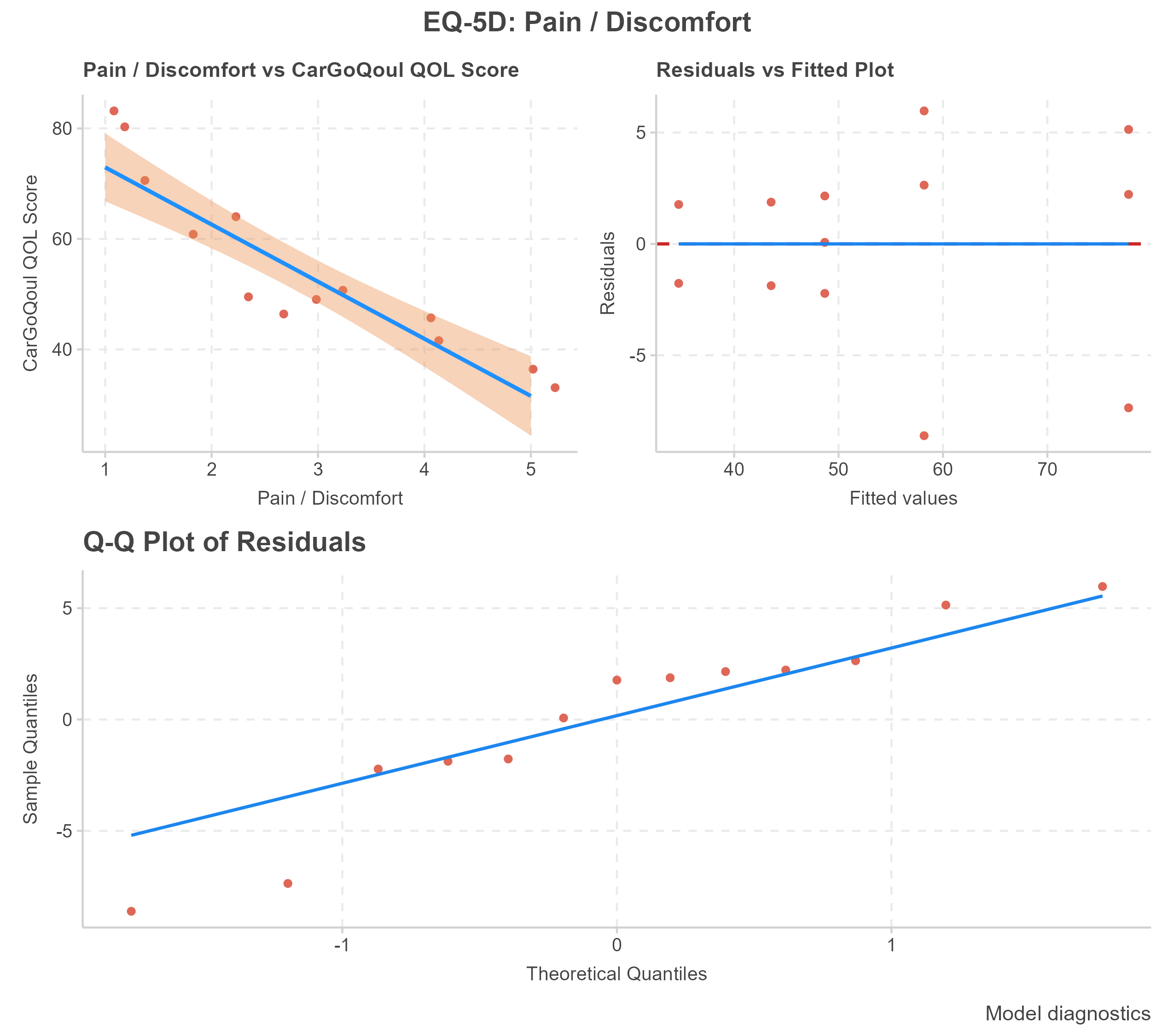


**
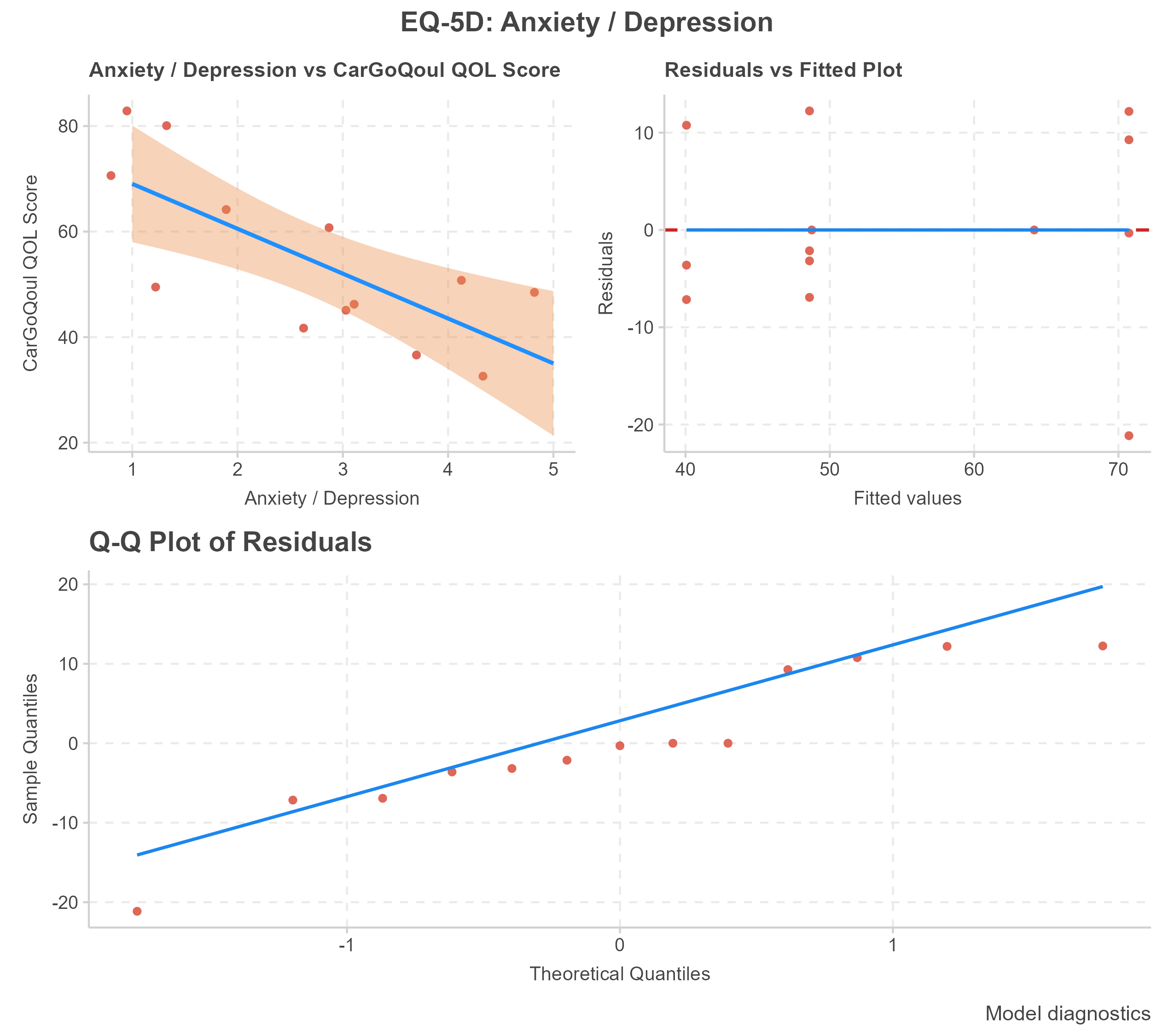
**

**Table S4. Characteristics of participant caregivers and matched non-caregivers**

| **Variable** | **Caregivers (n=13)** | | **Matched  non-caregivers (n=13)** | | **P-value** | **Statistical Test** |
| --- | --- | --- | --- | --- | --- | --- |
|  | **n** | **%** | **n** | **%** |  |  |
| **Gender** | | | | | >0.999 | Fisher's Exact Test |
| Female | 12 | 92.31 | 12 | 92.31 |  |  |
| **Age** | | | | | 0.898 | Mann-Whitney U Test |
| Median (IQR) | 44.92 (12) | | 44.46 (8) | |  |  |
| **Ethnicity** | | | | | 0.618 | Fisher's Exact Test |
| English / Welsh / Scottish / Northern Irish / British | 6 | 46.15 | 11 | 84.62 |  |  |
| Other | 2 | 15.38 | 2 | 15.38 |  |  |
| Missing | 5 | 38.46 |  |  |  |  |
| **Index of multiple deprivation (quintile)** | | | | | 0.823 | Mann-Whitney U Test |
| 1 (most deprived) | 1 | 7.69 | 1 | 7.69 |  |  |
| 2 |  |  | 4 | 30.77 |  |  |
| 3 | 6 | 46.15 |  |  |  |  |
| 4 | 3 | 23.08 | 4 | 30.77 |  |  |
| 5(least deprived) | 2 | 15.38 | 4 | 30.77 |  |  |
| Missing | 1 | 7.69 |  |  |  |  |
| **Region** | | | | | 0.510 | Fisher's Exact Test |
| East Midlands | 1 | 7.69 | 1 | 7.69 |  |  |
| East of England | 1 | 7.69 |  |  |  |  |
| London | 2 | 15.38 | 3 | 23.08 |  |  |
| North West |  |  | 3 | 23.08 |  |  |
| South East | 2 | 15.38 | 4 | 30.77 |  |  |
| South West | 2 | 15.38 |  |  |  |  |
| West Midlands | 1 | 7.69 |  |  |  |  |
| Yorkshire and The Humber | 2 | 15.38 | 1 | 7.69 |  |  |
| Wales | 1 | 7.69 | 1 | 7.69 |  |  |
| Channel Islands | 1 | 7.69 |  |  |  |  |
| **First symptom at date of birth** | | | | | 0.201 | Fisher's Exact Test |
| Yes | 6 | 46.15 | 2 | 15.38 |  |  |
| No | 7 | 53.85 | 11 | 84.62 |  |  |
| **Symptoms and co-morbidities** | | | | |  |  |
| Abdominal pain | 6 | 46.15 | 2 | 16.67 | 0.202 | Fisher's Exact Test |
| Abnormal heart | 2 | 15.38 | - | - | - | - |
| Diabetes | 2 | 15.38 | 1 | 8.33 | >0.999 | Fisher's Exact Test |
| Missing | 1 | 7.69 |  |  |  |  |
| **Count of symptoms and co-morbidities (per patient)** | | | | | 0.844 | Fisher's Exact Test |
| 0 - 5 | 2 | 15.38 | 3 | 23.08 |  |  |
| 6 - 10 | 3 | 23.08 | 5 | 38.46 |  |  |
| 11 - 15 | 4 | 30.77 | 2 | 15.38 |  |  |
| 16 - 20 | 2 | 15.38 | 2 | 15.38 |  |  |
| 21+ | 2 | 15.38 | 1 | 7.69 |  |  |
| Mean | 14.62 |  | 12.31 |  |  |  |
| Median (IQR) | 11 (9) |  | 10(8) |  |  |  |

**Table S5. Summary statistics of EQ-5D for non-caregivers**

|  | **No problem** | **Slight problems** | **Moderate problems** | **Severe problems** | **Extreme problems / Unable** |
| --- | --- | --- | --- | --- | --- |
| **Dimension** | **n (%)** | | | | |
| Mobility | 1 (7.69%) | 3 (23.08%) | 4 (30.77%) | 5 (38.46%) | 0 (0%) |
| Self-care | 4 (30.77%) | 3 (23.08%) | 4 (30.77%) | 1 (7.69%) | 1 (7.69%) |
| Usual activities | 2 (15.38%) | 3 (23.08%) | 5 (38.46%) | 2 (15.38%) | 1 (7.69%) |
| Pain / Discomfort | 0 (0%) | 2 (15.38%) | 4 (30.77%) | 5 (38.46%) | 2 (15.38%) |
| Anxiety / Depression | 2 (15.38%) | 6 (46.15%) | 2 (15.38%) | 2 (15.38%) | 1 (7.69%) |
